# Recovering distance information in spectral domain interferometry

**DOI:** 10.1038/s41598-018-33821-0

**Published:** 2018-10-18

**Authors:** Adrian Bradu, Niels Møller Israelsen, Michael Maria, Manuel J. Marques, Sylvain Rivet, Thomas Feuchter, Ole Bang, Adrian Podoleanu

**Affiliations:** 1 Applied Optics Group, School of Physical Sciences, University of Kent, CT2 7NH Canterbury, UK; 2 DTU Fotonik, Department of Photonics Engineering, Technical University of Denmark, DK-2800 Kongens Lyngby, Denmark; 3 Laboratoire d’Optique et de Magnétisme EA938, IBSAM, Univ. Bretagne Occidentale, C.S. 93837, 29238 Brest Cedex 3, France; 4 NKT Photonics A/S, Blokken 84, DK-3460 Birkerod, Denmark

## Abstract

This work evaluates the performance of the Complex Master Slave (CMS) method, that processes the spectra at the interferometer output of a spectral domain interferometry device without involving Fourier transforms (FT) after data acquisition. Reliability and performance of CMS are compared side by side with the conventional method based on FT, phase calibration with dispersion compensation (PCDC). We demonstrate that both methods provide similar results in terms of resolution and sensitivity drop-off. The mathematical operations required to produce CMS results are highly parallelizable, allowing real-time, simultaneous delivery of data from several points of different optical path differences in the interferometer, not possible via PCDC.

## Introduction

The key value of spectral (Fourier) domain interferometry (SDI) is its ability to encode spatial or temporal data into the spectrum at the interferometer output. The applications of SDI are wide-spread, encompassing spectroscopy^1^, astronomy^2^ and medical studies^3^ such as ophthalmology, where the latter is benefiting from the technique known as optical coherence tomography (OCT)^4^. Common to all techniques is the fact that the wider the spectrum the better the spatial/temporal resolution but the higher are the demands for the control of hardware dispersion effects. There are two modalities on transducing this information from the optical domain into electrical: spectrometer based interferometry where a broadband optical source is employed together with a spectrometer and swept source based interferometry, where a tunable (swept) laser is used and signal is delivered by a photo-detector. Both methods are characterized by nonlinearities in transferring the modulation of the optical spectrum into an electrical signal. Such nonlinearities lead to an irregular modulation (chirp) of the electrical signal read out by the spectrometer or the photo-detector while tuning the laser respectively. These nonlinearities can have two origins: the readout specificities^5^ (nonlinearities in the spectrometer or in the tuning of the swept source) and unbalanced dispersion in the interferometer and sample^6,7^. Unless this chirp is compensated for, after sophisticated linearisation procedures, a Fast Fourier Transform (FFT), applied to the electrical signal proportional to the spectra, leads to a wider and at the same time, reduced amplitude of the reflectivity profile peaks. Imperfections in these procedures become more obvious at larger optical path differences (OPDs) between the arms of the interferometer, and more pronounced as the spectral bandwidth of the optical source employed is increased.

In spectrometer based SDI instruments, the nonlinear distribution of optical frequencies over the linear array of the camera employed by the spectrometer is corrected by hardware solutions using either a prism after the diffraction grating^5^ or using a nonlinear electronic reading^8^. In swept source based instruments, nonlinearities in the tuning are due to the specific tuning element used. A hardware solution consists in using a k-clock. This requires a separate interferometer and a photo-detector faster than that needed in the measurement channel in sensing or imaging instrument in optical coherence tomography. However, especially when used for ultra-high resolution (UHR) instruments, these solutions complicate the hardware, require very careful adjustment, introduce losses and the linearity is not fully re-established. Due to their apparent simpler implementation, a software solution is often the method of choice to compensate for these nonlinearities^9,10^. The cost to be paid is the increased computational resources required by advanced resampling and linearisation algorithms that demand complex procedures involving the use of Graphic Processing Units (GPU)^11^, Field Programmable Gate Arrays (FPGAs)^12^ or optical computing^13^. Software solutions not involving resampling of data after acquisition were also proposed, namely, non-uniform discrete Fourier transform (NUDFT)^14,15^. A comparison of NUDFT vs FFT based methods has already been reported^15^ and demonstrated that the NUDFT methods can provide similar performance in terms of signal-to-noise ratio and axial resolution as the FFT based ones but are slower in terms of computational speeds. For this reason, these methods, based on building a Vandermonde matrix are often implemented by harnessing the computing capabilities of GPUs^16^.

In this paper we evaluate the performance of a newly introduced method to process the spectra at the interferometer output of a SDI device, that does not involve FFTs, Complex Master Slave (CMS). This method is compared with the conventional, widely used method based on FFT. Because the FFT method requires organisation of data in equal frequency slots, correcting algorithms have been devised. The method, proposed by Makita *et al*. in^9^, modified here and referred to as phase calibration with dispersion compensation (PCDC) is one of the most extensively used technique in OCT to linearise spectra, equally applicable to sensing and measurement of distances. This method is based on cancelling chirps in recorded interferograms based on the interferogram phase information, followed by resampling and correction for the unbalanced dispersion in the interferometer. After inferring the phase nonlinearities, data is resampled and then Fast Fourier transformed.

To avoid the disadvantages mentioned above, stemming from the use of FFT, the master/slave method was proposed by Podoleanu *et al*.^17^. This method is based on comparing raw acquired spectra with experimentally measured spectra (experimental masks) using a mirror, placed in the interferometer to create the OPD values where information from the sample is needed. This method was further improved to Complex Master Slave (CMS)^18,19^ wherefrom a reduced number of experimentally acquired spectra using a mirror, any number of masks are theoretically inferred.

By not performing FFTs, a radical change in data processing is established. A FFT simultaneously delivers amplitudes of back-reflections from all the points along the axial range investigated. This is especially useful in producing reflectivity profiles in depth (A-scans) in OCT. The development of long coherence length fast swept sources^20^ has allowed extension of OCT technology to topography of large objects and measurement of distances than can exceed several meters, with micrometer axial resolution^21^. In this case, the A-scan presents a single peak only, as in measurement of distances, useful in robotics, alignment, orientation and many other examples in industry. Such a method, with further development may become a competitor to more traditional methods of measuring large distances based on interferometry, achieving sub-micrometer axial resolution. Such a technique that allows large distance to be measured with sub-micrometer resolution is the frequency scanning interferometry (FSI)^22^. FSI measures the lengths of two interferometers at the same time and requires strict calibration of the wavelength using a gas absorption cell. By further extension of the tuning bandwidth of swept sources with long coherence length, one can achieve sub-micrometer resolution and can replace the complex FSI set-ups in the near future. What is radical as a difference between the FFT based PCDC and the comparison with masks based CMS is the fact that in the FFT, all resolved axial points are provided while CMS requires a processor for each such point. This may be initially seen as a disadvantage, but it is exactly this aspect that makes CMS more suitable to some applications than PCDC method. When only some points along the axial range are needed, CMS can deliver them directly, while when using PCDC, the calculation is executed over points that are not of any interest. Computation power has evolved tremendously in the last decade and parallel processing can be executed over the CPUs, to some extent with no need to recur to specialized means such as FPGAs and GPUs. From a different perspective, CMS is easier to implement than FSI. As detailed above, in FSI two cavities are measured at the same time, one as a reference and the other of unknown length. In CMS, comparison is also made between the unknown cavity and masks, put it differently, as virtual reference cavities.

In this paper, the theoretical background of both methods, CMS and PCDC is presented, followed by experimental assessment of their axial resolutions, sensitivity and processing time. To experimentally illustrate the capabilities of the two modalities, a UHR spectrometer based instrument equipped with a supercontinuum source was used. For such an instrument, correct OPD measurements are paramount to accurately produce depth resolved information.

All findings here have general validity. They are immediately applicable to any SDI system, employed for either accurate sensing of distances or for OCT. In continuation, reference to an A-scan should be understood in its general sense, either useful for extracting sample axial structure in OCT, or consisting in a single peak when measuring distance using reflection from a single mirror.

## Results

### Computation of the functions g and h

**Figure 1:**
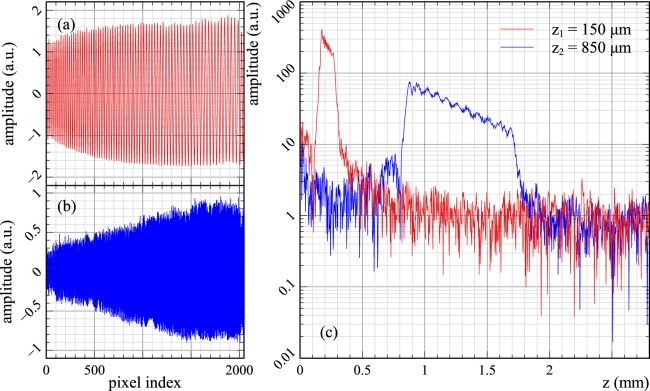
Examples of experimental spectra and their corresponding FFTs. (**a**) and (**b**): Experimental spectra collected for *z*_1_ = 150 *μm* and *z*_2_ = 850 *μm* used for calibration purposes. (**c**): FFTs of the spectra shown in (**a**) and (**b**).

As detailed in the Methods section, the information on the nonlinear dependence of the phase (*φ*) of the acquired signal at the interferometer’s output on the wavenumber (*k*), which in practice is the sampling coordinate, and on the dispersion left unbalanced in the interferometer can be described by using two functions *g* and *h* respectively. These functions, are related to the phase of the acquired signal via:

1 $$\phi (z,k)=g(k)z+h(k)+{\phi }_{rand},$$

where *φ*_*rand*_ takes into account possible random phase shifts between measurements, while *z* is the difference between the lengths of the interferometer’s arms. Computing these two functions, with high accuracy is paramount for both CMS and PCDC methods as they determine the performances of any SDI instrument. To this goal, for both methods, a flat mirror, *M*_*S*_ identical to *M*_*R*_, is used as sample, being placed in the focal plane of the telecentric lens SL (Fig. 13, in Methods, Experimental set-up subsection). This simulates a layer in the tissue sample in case of OCT or a corner cube in measuring of distances. Experimental spectra, *E*(*z*_1_, *k*) and *E*(*z*_2_, *k*), corresponding to two positions of *M*_*R*_, *z*_1_ and *z*_2_, are initially recorded for calibration purposes (*z*_1_ and *z*_2_ represent axial positions of a translation stage carrying *M*_*R*_, measured relative to the position where OPD = 0). Examples of spectra experimentally collected for *z*_1_ = 150 *μm* and *z*_2_ = 850 *μm*, are presented in Fig. 1(a,b).

As illustrated in Fig. 1(c), the FFTs of these spectra display wide peaks, respectively spanning from hundreds of microns to nearly 1 mm due to the distribution of the wavenumbers along the pixels of the camera and unbalanced dispersion in the interferometer. Using the spectra collected for *z*_1_ = 150 *μm* and *z*_2_ = 850 *μm*, parameters for the two methods, PCDC and CMS, according to the procedures presented in the Methods can be computed. These parameters represent the nonlinear coefficient *g* and unbalanced dispersion *h* for the PCDC and CMS respectively: *g*_*PCDC*_, *h*_*PCDC*_, *g*_*CMS*_ and *h*_*CMS*_.

**Figure 2:**
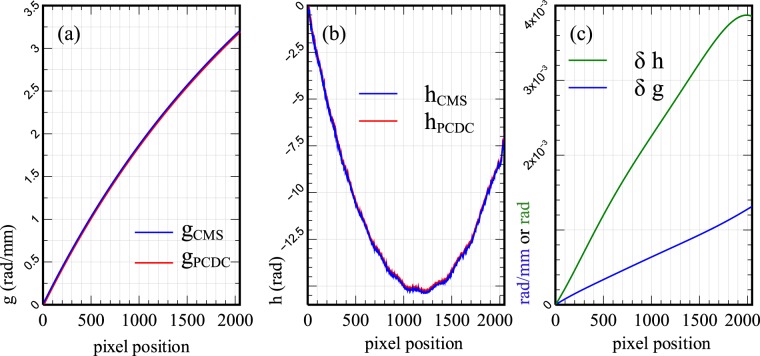
Functions *g* and *h*. (**a**) Function *g*, (**b**) function *h*. The two functions were calculated for both PCDC (red) and CMS (blue) cases, using the procedures presented in Methods (the two experimental spectra presented in Fig. 1(a,b) were used). (**c**) *δg* and *δh*: differences between the parameters *g* and respectively *h* measured at t = 0 and t = 5 weeks using CMS only (normalised with respect to their corresponding first pixel value).

In PCDC, the effect of the random phase shift is neglected (*φ*_*rand*_ = 0). As a result, for a given set the two experimental spectra, the values of the parameters *g* and *h* differ slightly from those obtained using CMS. This is illustrated in Fig. 2, where the two parameters are computed for each pixel position of the camera using the two methods. To distinguish between the red (CMS) and blue (PCDC) curves, the plots were intentionally shifted slightly in the vertical direction. Numerically, we found out that the difference between the *h*-values computed using the two methods can be quantified as $RMS(|{h}_{PCDC}-{h}_{CMS}|)=2.57\times {10}^{-14}$ rad, while the difference between the two *g*-values as $RMS(|{g}_{PCDC}-{g}_{CMS}|)=1.64\times {10}^{-15}$ rad/mm.

Irrespective of the moment in time the calibration was performed, approximately the same RMS value between each of the functions *g* or *h* computed with PCDC and CMS respectively was found. When comparing the parameters g(t = 0) to g(t = 5 weeks), and h(t = 0) to h(t = 5 weeks) we found out that the variations *δg*_*CMS*_ = *g*_*CMS*_(5*weeks*) − *g*_*CMS*_(0) and *δg*_*CMS*_ = *g*_*CMS*_(5*weeks*) − *g*_*CMS*_(0) are very small. Typical examples of such variations are shown in Fig. 2(c). For the case of PCDC, the amplitude of these variations is larger by a factor of 2–3 than for the CMS. As the parameters *g* and *h* produced by the 2 methods (at a given time) do not differ too much, it is expected that the axial resolutions computed just after calibration do not differ substantially.

The deviation from a linear dependence of the parameter *g* with the pixel position can in fact be used to quantify the nonlinearity of the system, while the deviation of *h* from a constant indicates that there is a certain amount of dispersion left unbalanced in the system. Ideally, *g* should be proportional to the wavenumber. An increase with pixel number discloses the geometry of the spectrometer, with red edge towards the low pixel number and blue edge towards high pixel number. A rotation of the grating may lead to *g* decreasing with pixel number.

The plots presented in Fig. 2 were produced using the experimental spectra presented in Fig. 1(a,b). Using other pairs of experimental spectra, recorded at various other axial positions of the reference mirror than those shown in Fig. 1, similar shapes for the parameters *g* and *h* result. Small differences in *g* and *h* obtained for different sets of experimental spectra obtained at various OPDs together with the fact that the reflectivity profiles are calculated differently (interpolation vs. multiplication), can potentially lead to differences between the axial resolutions obtained with the two methods. To investigate this issue, pairs of experimental spectra were recorded and used to produce A-scans according to Eqs 4 and 8 and the full-width-at-half-maximum (FWHM) of the peaks obtained was evaluated.

**Figure 3:**
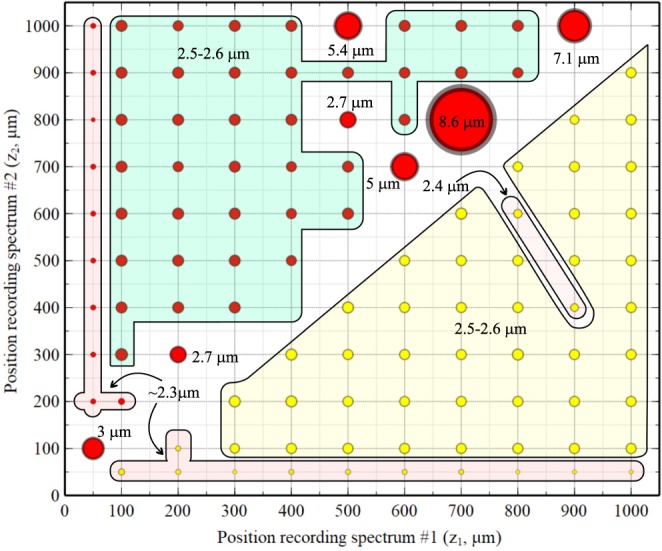
Axial resolutions obtained using various pairs of spectra produced using Eqs 4 (PCDC) and 8 (CMS). The values obtained using PCDC are represented by red circles, while those obtained with CMS by yellow circles. The size of the circular symbols indicates the resolution obtained.

The values of the axial resolutions hence obtained are summarized in Fig. 3. A sequence of *i* = 1…11 experimental spectra were recorded for *z*_*i*_ = 50, 100, 200, 300, 400, 500, 600, 700, 800, 900 and 1000 *μm* and then pairs of these spectra were used to calculate the parameters *g* and *h* according to the two methods. For each case, the axial resolution was evaluated by calculating the FWHM (Gaussian fit) of the peak of an A-scan generated for the experimental spectrum recorded at *z* = 1.0 mm. For representation of axial resolution variation, circular symbols of different diameters are shown in Fig. 3, where for better illustration their size is nonlinearly dependent on the actual computed axial resolution. The size of the red symbols represent values of the axial resolutions obtained using Eq. 4 (PCDC), and the yellow ones values of the axial resolutions using Eq. 8 (CMS).

A first observation resulting from analysing Fig. 3, is that the choice of the two experimental spectra has some influence on the axial resolution obtained. However, the spread of resolution values is narrower for CMS, where they vary between 2.3 and 2.6 *μm*, with most of results around 2.5–2.6 *μm*. In most of the cases, PCDC produces values of the resolution in the same range, 2.3 to 2.6 *μ*m with most of the values around 2.5–2.6 *μ*m as in the CMS case. However, in some cases (12.7% of the points presented here), PCDC fails to produce a good axial resolution. The combination of experimental spectra producing large values of the resolution intervals seems to involve spectra recorded for large, not very different values of *z*. On one occasion (*z*_1_ = 50 *μm* and *z*_2_ = 100 *μm*), PCDC also provides a resolution larger than 2.6 *μ*m. As the span of the resolutions’ values does not typically exceed 0.3 *μ*m, it seems that in most of the situations the choice of the two experimental spectra used for calibration purposes is not particularly important. Irrespective of the choice, successful compensation is achieved for the effects of nonlinearities and reading the spectra and of the unbalanced dispersion. Yet, for the PCDC case, some attention needs to be paid, as some combinations can lead to a wavenumber distribution for which the cubic spline interpolation fails.

**Figure 4:**
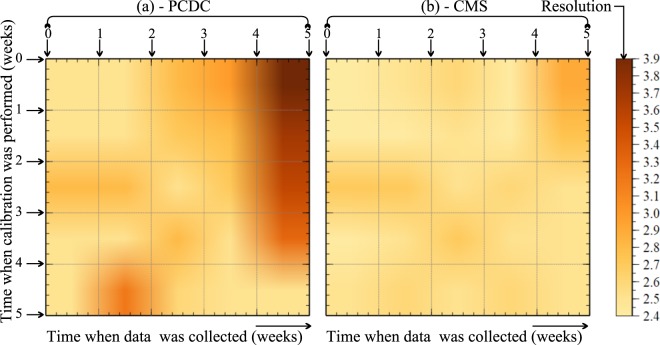
Axial resolutions produced using calibration spectra recorded over time. Vertical axis: time when the calibration spectra were recorded. Horizontal axis: time when resolutions were computed. Each color encodes a certain axial resolution according to the colormap displayed on the right.

To investigate the effect of the random phase shift, pairs of experimental spectra were recorded for calibration purposes over a period of five weeks and used to compute the axial resolutions. In Fig. 4, along the vertical axis we display the time when calibration spectra were recorded, while along the horizontal axis the time when the spectrum is recorded and axial resolution computed. Along both axes, one unit represents one week. All calculations are performed for *z* = 1.0 mm and color coded, from yellow to brown, where yellow corresponds to an axial resolution of around 2.4 *μ*m, while brown to 3.9 *μ*m (right side of Fig. 4).

As expected, when the moments for experimental spectra acquisition and calibration are close to each other, the best values of the axial resolutions are obtained. These are shown along the diagonal of the images in Fig. 4, from the top left to the bottom right, of around 2.4 *μ*m, for both cases, PCDC and CMS. However, when the time interval between the calibration and the imaging steps increases, both methods suffer. As an example, when the calibration and the imaging steps are both done at *t* = 0, the axial resolution is around 2.4 *μ*m, whilst when the same experimental calibration spectra are used to produce images at *t* = 5 weeks, the values of the resolution deteriorate to around 3.8 *μ*m for the PCDC and 3.0 *μ*m for CMS. Random fluctuations, of small amplitude, in the axial resolution are observable in Fig. 4 during the 5 weeks period, which could be attributable for example, to the fluctuations in the phase of the light emitted by the supercontinuum optical source, changes in the shape of spectra recorded by the linear camera, micrometric mechanical shifts, etc. However, the CMS method seems to be slightly more tolerant to these temporal fluctuations. For our particular instrument, these random phase fluctuations seem to lead to failures of the interpolation procedure.

**Figure 5:**
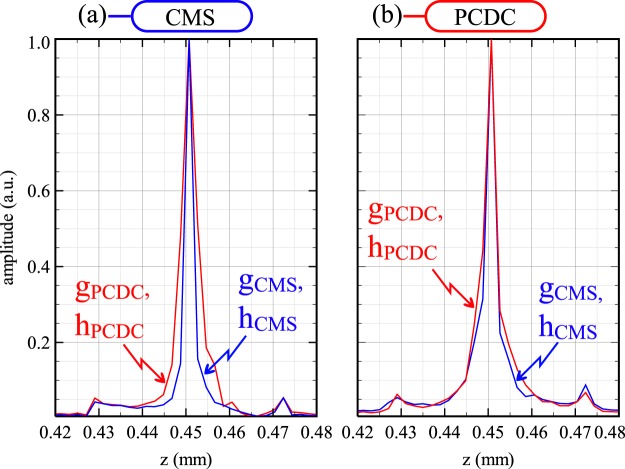
Normalized axial reflectivity profiles using: (**a**) CMS and the (**b**) PCDC methods. The red curves were produced using the *g*_*PCDC*_ and *h*_*PCDC*_ as obtained by PCDC, while the blue curves were produced using *g*_*CMS*_ and *h*_*CMS*_ as obtained by CMS.

Even small differences between the parameters *g* and *h* produced by the two methods are sufficient to alter the axial resolution. To illustrate this effect, we have used parameters *g*_*PCDC*_ and *h*_*PCDC*_ to produce both a PCDC and a CMS axial reflectivity profile, and parameters *g*_*CMS*_ and *h*_*CMS*_ to produce both a PCDC and a CMS profile as shown in Fig. 5, where:

- CMS profiles were produced using parameters: *g*_*CMS*_ and *h*_*CMS*_ as computed by the CMS method (blue) and *g*_*PCDC*_ and *h*_*PCDC*_ as computed by PCDC (red).
- PCDC profiles were produced using parameters: *g*_*PCDC*_ and *h*_*PCDC*_ as computed by the OCDC method (red) and *g*_*CMS*_ and *h*_*CMS*_ as computed by CMS (blue).

It is quite obvious that swapping the *g* and *h* parameters between procedures can lead to a significant deterioration of axial resolution. The use of theoretical inferred spectra not corrected for the random phase (i.e. the PCDC spectra) leads to worse axial resolution in CMS (Fig. 5(a) - red curve), while the elimination of the random phase when the calibration vector is produced leads to a slight improvement of the axial profile shape in PCDC (Fig. 5(b) - blue curve).

### Sensitivity drop-off and axial resolution

An important parameter in measuring distances using both spectrometer and swept source based SDI methods is the axial range possible to be targeted, i.e. how far away the corner cube can be placed from the beam-splitter to still be able to generate a sufficient strength interference signal. This parameter is also important in OCT, determining the imaging range in the tissue. Such a measurement is described by the sensitivity drop-off, that shows reduction in the interference strength with OPD. The cause for reduction is the “dynamic coherence length” of the two interference waves^23^. In a spectrometer based instrument, this is determined by the coherence length of waves after diffraction on the grating combined with the spectral capability of the camera in sampling the spectrum^24^ while in a swept source based one by the inverse of dynamic line-width^21^.

**Figure 6:**
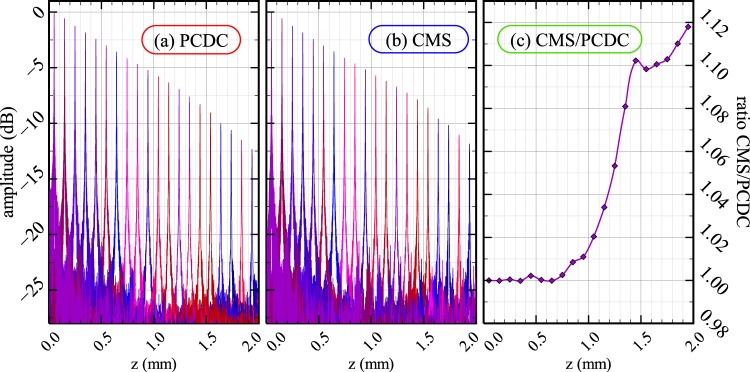
Normalized sensitivity drop-offs produced using the (**a**) PCDC (**a**) and (**b**) CMS methods. (**c**) Ratio between the amplitudes in (**a**) and (**b**).

Sensitivity drop-off is measured by placing the mirror *M*_*s*_ at increased distances *z*. Normalized sensitivity drop-offs (with respect to the first A-scan, recorded at *z* = 50 *μ*m), are produced using PCDC in Fig. 6(a) and using CMS in Fig. 6(b) respectively. To produce these graphs, for calibration purposes, experimental spectra recorded at *z*_1_ = 150 *μ*m and *z*_2_ = 850 *μ*m were used. Although the decaying slopes in Fig. 6(a) and Fig. 6(b) are similar, some sensitivity advantage is seen for the CMS at large OPD values. To illustrate this slight improvement, the ratio of graph amplitudes in Fig. 6(b) and (a) is represented in Fig. 6(c), where for instance at 2 mm, CMS is 12% better than PCDC. For this particular instrument, for both methods, the sensitivity drops by around 5.5 dB at *z* = 1 mm and 11.5 dB at *z* = 2 mm in respect to the sensitivity measured at *z* = 50 *μ*m.

**Figure 7:**
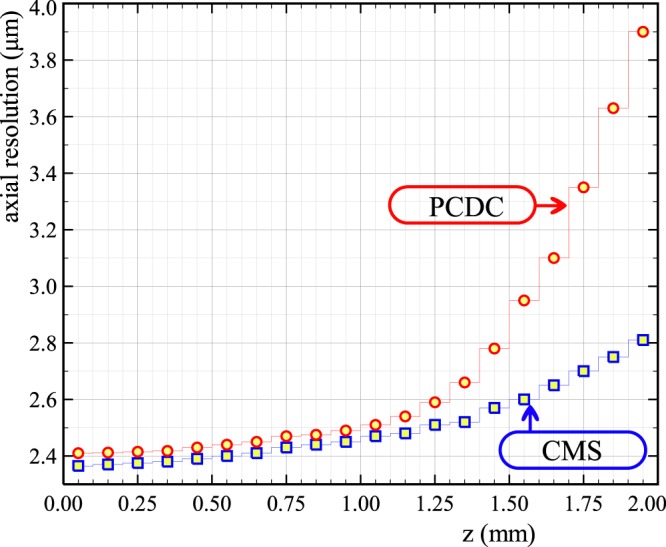
Axial resolutions computed using PCDC and CMS.

Figure 7 shows the FWHM axial resolution measured using a Gaussian fit for each A-scan peak in Fig. 6(a) and (b). No data apodization was used to produce Fig. 7. In both cases, the resolution worsens with depth. This is due to the limited number of pixels available for the camera utilized by our spectrometer (2048). A large bandwidth per each camera pixel translates into equivalent reduction of the dynamic coherence length of the interfering waves. A poor sampling of spectrum with a reduced number of camera pixels leads to a spline interpolation failure of the high frequency spectra, as documented by Yun *et al*.^25^. This affects both PCDC procedure and the multiple signal comparison operations practised by the CMS.

Up to around *z* = 1.2 mm, both methods show similar values of the axial resolution in the region of 2.4–2.6 *μ*m for PCDC and 2.3–2.5 *μ*m for CMS. Beyond this point, the axial resolution deteriorates faster when using PCDC. At z = 2 mm, the axial resolution as measured using the CMS method is around 1 *μ*m better than that provided by PCDC. This could be explained by the fact that the mathematical operations used to compute the axial reflectivity profiles, spline interpolation and FFT for PCDC are less tolerant to amplitude reduction with OPD than the comparison operation of spectra via multiplications of 1D arrays for CMS. Possibly, the significantly degraded signal strength for the higher frequency modulation band edge of the chirped spectrum at large OPDs (Fig. 1(b)), can further introduce an error in the step of data interpolation. Therefore, Fig. 7 quantifies the information loss in the act of interpolating spectra, when approaching the Nyquist frequency in combination with inconsistent signal strength across the band resulting in a resolution loss of 1 *μ*m.

To produce Figs 6 and 7, which show a slight benefit of using the CMS over PCDC in terms of signal-to-noise ratio and axial resolution, data was oversampled by a factor *α* = 4. The oversampling was produced either by zero padding the spectrum before FFT (PCDC case) or by generating a higher number of theoretically inferred spectra (CMS case). Vergnole *et al*.^15^ reported that both the NUDFT and FFT based method using a cubic spline interpolation with data oversampled by a factor *α* = 3 lead to similar results in terms of signal-to-noise ratio and axial resolution for any axial depth (in this case(Vergnole *et al*.), the oversampling was performed on the spectra). Methods of improving the sensitivity at depth were reported^26,27^, however, for the present study no digital technique has been used to improve the sensitivity at depth for neither of the techniques. The fact that CMS behaves slightly better at depth than PCDC may be considered as due to imperfections in the interpolation technique employed here for PCDC (cubic B-scan interpolation). Vergnole *et al*.^15^ have showed that, for up to 2 mm depth, when using a spline cubic interpolation (PCDC), sensitivities as good as those provided by NUDFT can be achieved, irrespective of the oversampling factor. Therefore, within the axial range achievable with our instrument, we can assume that a comparison of CMS with NUDFT can be made. The reason why CMS should perform better at larger depth than NUDFT is that NUDFT does not take into account the dispersion effects in the interferometer. This is particularly important as our findings are on a ultra-high resolution instrument using a supercontinuum optical source whose spectrum spans over 400 nm, providing axial resolutions 8 times better than those of Vergnole *et al*.’s instrument.

### Axial reflectivity profiles from multiple scattering centres

So far we referred to a single mirror reflector, such as used in sensing of distances or in calibration of OCT signals. We illustrate further the behaviour of the two methods faced with a succession of scattering centres in tissue, typical for OCT investigations. Using the instrument shown in Fig. 13, equipped with a galvanometer scanner, various samples were imaged, and cross-section images (B-scans) were produced.

**Figure 8:**
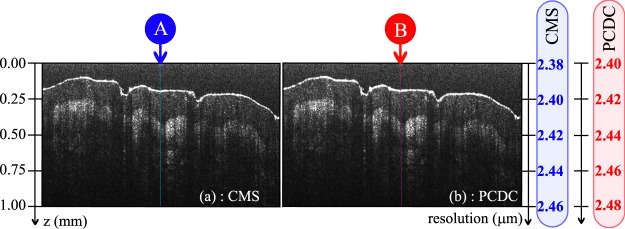
Cross-section images of a thumb obtained using CMS (**a**) and PCDC (**b**). The left vertical axis is depth *z*, the right vertical axes are the axial resolutions evaluated in air using both methods.

Figure 8 shows, side by side, a typical example of cross-sectional images of the human thumb, produced using PCDC and CMS, respectively. Another pair of images showing the junction nail/skin is also provided (Supplemental Figure S1) as well as pairs of A-scans extracted from these images at positions labelled A1, B1, A2 and B2). To reduce spectral leakage, the acquired experimental spectra (PCDC) or the theoretical inferred ones incorporated in matrix M (CMS) were both subject to apodization via a Gaussian window, as detailed in the Methods. This minimises the amplitude of the aliasing side lobes. In addition, the experimental spectra were oversampled again, by a factor *α* = 4 when using PCDC, so the number of axial points in each A-scan was 4096. To obtain the same number of axial points when using CMS, a number of 4096 theoretical spectra were inferred.

As expected, both images are of similar quality in terms of resolution and sensitivity. In Fig. 8, the left vertical axis is *z*, while the right vertical axis is the axial resolution as measured in air using CMS and PCDC respectively using the values in the graphs in Fig. 7. As the values of the resolutions obtained with CMS are typically only 0.1–0.2 *μ*m better than those obtained using PCDC it is not possible to clearly distinguish differences between the two images.

**Figure 9:**
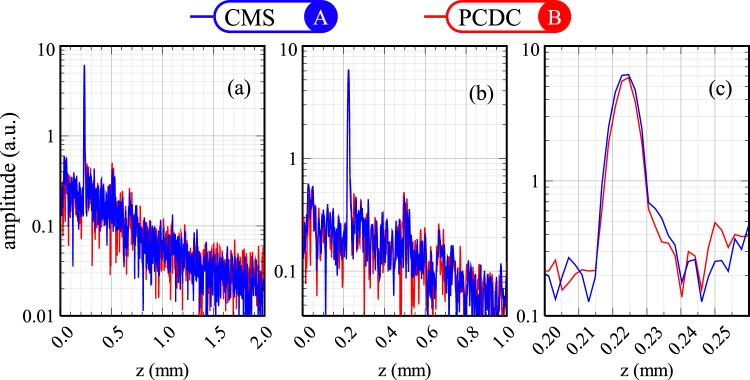
A-scans produced using PCDC and CMS when the sample is the human thumb over: (**a**) the entire axial range from 0 to 2 mm; (**b**) 0–1 mm; (**c**) narrower range (0.22–0.30 mm). Data were extracted from the images shown in Fig. 8 at the positions marked by a blue dashed line (CMS) and red dashed line (PCDC).

From Fig. 8, A-scans were extracted from the CMS image (position A) and PCDC image (position B) respectively. Both A-scans were extracted from the middle of the two images (blue and red lines respectively in Fig. 8). The two A-scans are presented over the whole axial range available (2 mm, Fig. 9(a)), over a shorter range of 1 mm (Fig. 9(b)) and also over a 65 *μ*m axial range (Fig. 9(c)). As expected, both profiles are similar in terms of resolution and strength.

### Benchmarking

A last element of comparison between the two methods that we looked at was the capability of the two methods to produce sequences of A-scans (B-scan) in real-time. For this purpose, the time to produce a B-scan was computed using a LabVIEW 2017 (National Instruments, Austin, Texas) program installed on a PC equipped with an Intel i7-7800X @ 3.5 GHz (6 cores, 12 threads of execution) CPU and 16 GB RAM.

**Figure 10:**
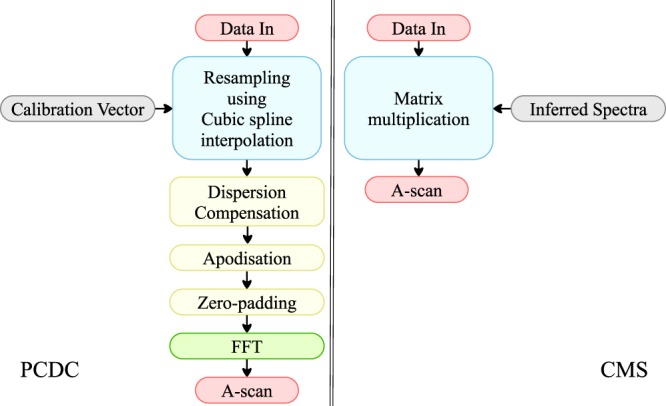
Simplified flowchart illustrating the benchmarked mathematical operations performed after data acquisition.

A simplified flowchart detailing only the benchmarked mathematical operations performed after data acquisition is shown in Fig. 10. A detailed presentation of the mathematical operations performed before and after data acquisition can be found in the Methods section. When using PCDC, at the calibration step, a calibration vector $\hat{k}$ is produced. This vector is then used to resample the data using a cubic spline interpolation. The resampled data is then sequentially subject to apodization, correction for unbalanced dispersion, zero-padding and finally FFT. When using CMS, the only operation required is the multiplication of the matrix T containing theoretically inferred spectra provided by the calibration step by the acquired data vector. In this case, the apodization of the inferred spectra is done at the calibration step. Zero-padding is also not required at this stage as the number of axial points is determined by the size of matrix T generated at the calibration step.

**Figure 11:**
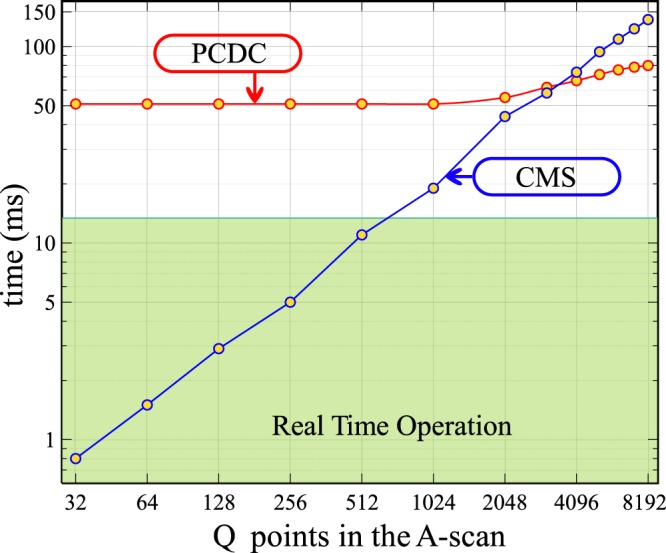
Time to acquire and process *P* = 1024 spectral acquisitions using PCDC (blue) and CMS (red) vs. the number of axial points targeted axially.

Let us consider a number *P* = 1024 of spectral collections. As the linear camera delivers spectra at a frequency of 76 kHz, the acquisition time of *P* spectra is 13.4 ms. This corresponds to a distance measurement using an average of *P* spectra or to the production of a cross section B-scan image in OCT consisting in *P* lateral adjacent A-scans. The instrument will operate in real-time when the time to process data is shorter than the data acquisition time. In Fig. 11, the time to produce *P* A-scans vs. the number of points *Q* targeted axially is presented. For PCDC, *Q* is adjusted by zero padding each signal, after data resampling, before FFT. When employing CMS, *Q* is equal to the number of masks produced at the calibration step. Using FFT, PCDC delivers all *Q* points over the whole available depth range (i.e. in our case over 2 mm).

As PCDC is based on data resampling, followed by FFT, while CMS on matrix multiplication, the number of operations involved by the two techniques are different, so it is expected that one of them to be faster than the other as illustrated in Fig. 11 depending on the number *N* of points the spectra is sampled into and the number *Q* of points targeted in the axial reflectivity profile. The PCDC based strategy is obviously faster in terms of producing a reflectivity profile, when no data preparation is required before FFT. Indeed, in principle, if each spectrum is sampled into *N* points, PCDC requires *Nlog*_2_*N* operations to produce an axial reflectivity profile, while CMS *Q*(2*N* − 1) operations, where *Q* is the number of points to be computed for each axial profile. For PCDC, this number is *N*/2. However, as mentioned above, CMS offers the freedom to provide reflectivity profiles over subintervals of the axial range, using a lower number of points than *N*/2, hence for values of *Q* sufficiently small, CMS may perform faster. To produce Fig. 11, we considered the situation where *N* = 2048 (number of pixels on the camera). By oversampling data by a factor *α* = 3, for example, A-scans containing a number of 3072 axial points were produced using PCDC. With PCDC, the same number of axial points is generated at the calibration step by theoretical inferring the corresponding number of spectra. According to our benchmarking, both methods, produce 1024 axial profiles, each of 3072 points, in around 60 ms.

When data is resampled using a cubic B-spline interpolation, Vergnole *et al*.^15^, found that for an oversampling factor *α* = 3 of the experimental spectra, the FFT procedure is around 7 times faster than the matrix multiplication based NUDFT. The oversampling of the spectrum is performed after data acquisition, for both methods, PCDC and CMS. After oversampling, to produce an A-scan, a FFT of the oversampled experimental spectrum (PCDC) or a multiplication between the oversampled experimental spectrum and the theoretical inferred ones, also oversampled (CMS) needs to be executed. As in both cases, data must be resampled, the PCDC method is a clear winner in terms of processing time. CMS can compete with PCDC only when a low number of axial points *Q* is required. Our benchmarking indicates that CMS can be faster than PCDC only when *Q* is less than 128.

As recognised in the same paper, comparing computational times from different publications is a daunting task. The benchmarking provided in^15^ was produced on a computer equipped with an old processor, while no optimisation technique of matrix multiplication was employed. In the current manuscript, the full capabilities of LabVIEW of parallelising the data flow were used in both cases. The most time expensive operation in PCDC is the cubic spline interpolation. This was implemented using the LabVIEW’s Spline Interpolation Virtual Instrument. To perform the matrix multiplication, we took advantage of the LabVIEW’s Multicore Analysis and Sparse Matrix Toolkit.

As it can be observed in Fig. 11, it is impossible for our hardware configuration to produce real-time measurements of distances or cross-sectional images using PCDC. The whole sequential process of operations involving apodization, spline interpolation, zero padding and FFT applied to each of the *P* spectra acquired, takes around 50 ms, while data is acquired in 13.4 ms. With CMS, the real-time operation is possible for a limited number of points *Q*. The apodization is done at the calibration stage, by altering the shape of the masks, while after data acquisition only a multiplication of matrices is required. Thus, if less than *Q* = 700 axial points are required, the system can operate in real-time. With PCDC, there is no way to define an axial region of interest (AROI) where the reflectivity profile is produced exclusively from. For CMS, such an AROI can be defined hence, if necessary a lower number of axial points in the A-scan can be computed. This makes real-time operation of the instrument possible. For our given set-up, the achievable axial resolution is around 2.5 *μ*m. If we assign the 2.5 *μ*m to each pixel along depth in the A-scan, a maximum AROI of 1.75 mm can be produced in real-time using CMS.

Let us now restrict the AROI to the range within which the resolution is that theoretically inferred. If we suppose a Gaussian shape of the spectrum emitted by the supercontinuum source centred at 1270 nm, FWHM of 400 nm, the theoretical optical axial resolution is ≈1.8 *μ*m. With the same evaluation as above, then we are limited at processing an AROI of 1.26 mm in real-time. For such an AROI, the *P* A-scans are either averaged for distance measurements or assembled in a B-scan for OCT.

To produce Fig. 11, the number of axial points was adjusted by zero padding in the Fourier domain (PCDC) or generating different numbers of theoretically inferred spectra (CMS). Here, we used the zero padding method due to its popularity. Other faster methods of interpolation exist. However, it must be noted that when no interpolation is performed, the CMS is faster than PCDC by at least a factor of 2, so the choice of the interpolation method for PCDC is not relevant. This is illustrated in Fig. 11 (Q = 1024). For the CMS case, the execution time increases faster with *Q* than in the PCDC case, for which reason, for high *Q* values, CMS does not bring benefits in terms of execution time over PCDC, not even when PCDC uses zero padding for interpolation.

## Conclusions

Two approaches of decoding spectra in spectral domain interferometry are compared, delivering reflectivity profiles useful for distance measurements and OCT: a novel method, involving a calculation for each distance (depth of interest) (CMS), and the FFT based conventional method (PCDC). The mathematical apparatus behind each technique was presented, and experiments were conducted to compare the accuracy in distance measurement in sensing and depth representation in OCT using both methods implemented in a spectrometer based instrument equipped with a supercontinuum optical source. According to the theory, both methods should converge to similar conclusions. However, when dealing with experimental data, although the results obtained with the two methods are in most cases similar, subtle differences were noticed. The PCDC relies on interpolation of data and FFT whilst the CMS relies on multiplying electrical signals proportional to the spectra acquired.

With both techniques we were able to produce high axial resolution distance measurements and A-scans with better than 2.5 *μ*m and of similar sensitivity. However, when considering the stability of the calibration and the rate at which UHR axial measurements are produced, CMS came as the technique of choice for the following reasons:

- Tolerance to choice of OPD values for calibration. CMS’ axial resolutions are not as dependent on the choice of the experimental spectra employed at the calibration stage as PCDC.
- Calibration stability over time. CMS copes better with phase fluctuations. The same spectra used for calibration purposes can be used over a longer period of time.
- Time required for an AROI shorter. For a reduced number of pixels along the axial range, CMS can operate in real-time. For OCT, 1 mm axial range may be sufficient while maintaining the axial resolution theoretically expected. Even more, a sparse OCT image can be quickly produced, let us say for 10 mm. A sparse measurement of distances can also be produced fast if speed is more important than axial resolution.
- Flexibility. The demands on the computational resources can be adjusted in accordance with the axial range and resolution targeted, rendering the whole process of distance measurements and OCT imaging more efficient.
- Constant distance/depth measurement. Using a single mask, CMS can preferentially identify reflectors at a desired distance or produce constant depth OCT images (*en-face*), corresponding to points at the distance or respectively depth matching the OPD used to produce that mask. This may impact procedures of seeking reflectors positioned around a point, or producing topography of a single layer objects, or *en-face* OCT images.
  Despite the advantages that the CMS approach offer, some drawbacks of the method still exist:
- At the calibration step, the mathematical complexity of inferring theoretical spectra to build matrix T is apparently higher as it involves the use of low level computational routines. When using the NUDFT, the construction of the Vandermonde matrix is a simpler task as it is provided by LabVIEW as a virtual instrument while MATLAB provides it as an inbuilt function, etc. However, through simple steps, clearly described in our papers^18,19^ a ready to use toolkit can potentially by developed.
- After data acquisition, parallelisation of data processing is a must. This has to be done using optimised low-level routines for performing common linear algebra such as BLAS (Basic Linear Algebra Subprograms) or other tools which take advantage of the multicore processors such as LabVIEW’s Multicore Analysis and Sparse Matrix Toolkit that we used in this report.
- Although CMS can operate in real-time for a low number of axial points *Q*, for large dimension of matrix M, CMS not only incapable of real-time operation but performs slower than its PCDC counterpart. This may be the case in some particular situations as for example in (i) spectrometer-based interferometry when cameras using large number of pixels aimed at increasing the axial imaging range, (ii) swept-source interferometry when the combination between the digitizer’s sampling rate and the tuning speed of the laser leads to an increased number of points each spectrum is digitized into, (iii) there is need of large number of axial points *Q* in the A-scan.

Essential in comparing the two methods presented here is the peculiarity of CMS of not relying on FFTs. Replacement of a single FFT operation in PCDC with multiple comparison operations in CMS determines a radical difference between the two methods. CMS was initially motivated by the need to directly deliver, *en-face* OCT images, with no need to assemble the volume of A-scans. Soon it was realised that other advantages exist, such as tolerance to the spectrum chirp. This study focuses into another direction opened by CMS, that of metrology of measuring distances. There are however other related potential avenues that are worth being investigated, such as customised processes for specifically targeted AROIs and sparse delivery of data that cannot be approached using a Fourier transform.

For the current study, no digital technique was used to enhance the sensitivity at depth. While popular interpolation methods such as zero-order (nearest neighbour), first-order (linear) and third order (spline) can boost sensitivity at large depth on the expense of the calculation speed of producing reflectivity profiles (by using a large oversampling factor *α*), other methods exist where small values of *α* are required to achieve a good sensitivity at depth. Such an example is the method suggested in Vergnole *et al*.^15^, who demonstrated that by resampling through convolution using an optimized Kaiser-Bessel function, the sensitivity at depth is enhanced when an oversampling factor *α* as small as 1.2 is used. Here, when using CMS, the oversampling is achieved by increasing the number of OPD values used to calculate the theoretically inferred spectra. This involves matrix multiplications that demand less resources than spline interpolation, hence practically less penalty on the computing time. This is also accompanied by an increase in the sensitivity decay with depth. This leads to an enhancement of the sensitivity at depth over PCDC without any obvious penalty on the computation time.

An application that would tremendously benefit from the capability of CMS to provide real-time imaging is that of high resolution Optical Coherence Microscopy (OCM)^28^. This uses a high numerical aperture interface optics that leads to very narrow confocal profile, hence a very limited axial range. In order to adapt spectral domain OCT to high numerical aperture OCM, a technique was reported based on Gabor filtering^29,30^ where acquisition is repeated for several focus positions, that shifts the confocal gating profile incrementally through the sample depths. If PCDC was used, large portions of the OCT axial range within each A-scan are discarded, as outside the narrow confocal profile. Put it differently, PCDC is highly inefficient when combined with Gabor filtering that requires repetition of acquisition for each new position. With CMS, data processing is faster, as we can limit the computation to points from within the depth of focus^31^.

If information from a single depth is needed, this requires a single multiplication operation. *Q* = 1 is the extreme case of a range of *Q* values where CMS is faster, as shown in Fig. 11. This can refer to the detection of axial position, if a mirror crosses the depth of interest, as determined by one of the theoretically inferred spectra, used as calibration. There are various other applications where a single peak is needed, as for instance in tracking the position of a moving mirror (such as cornea in an axially moving eye). In this case, the relative position of the cornea from an initial reference position is needed. This can be manually identified and optical path difference zeroed, leaving the tracker to monitor the axial position from this reference position, normally within a fraction of the axial range. In practice, if the tracking range of axial positions of the moving mirror (cornea) can be restricted to within the range of *Q* values where CMS is faster, according to Fig. 11, then CMS is the method of choice. If the initial position of the mirror is not known, or if the tracking interval reaches the extension of the axial range, then this would require engaging all N reference spectra, to cover the whole axial range, in which case the FT based method may be faster.

In terms of parallel processing, we have shown here that current CPUs are capable of processing CMS signals with no need to resort to FPGA or GPUs. However, the CMS potential is there, where the numerous comparison operations needed can be performed on FPGAs and GPUs, to improve the real-time processing making the CMS efficient even in those cases where CMS was deemed slower than PCDC (according to discussion around Fig. 11) where CPU only was used. A spectrometer based SDI instrument was employed here, but all results are immediately applicable to any spectral domain interferometry system.

## Methods

### Methods for obtaining axial reflectivity profiles

To produce an A-scan, *A*(*z*)^*chirped*^, of the sample under investigation, the integral of the product between an experimentally acquired spectrum *E*[*φ*(*z*, *k*)], obtained by interfering light from the sample and reference arm of the interferometer and the kernel function *e*^−*jkz*^, is calculated. Here, *φ*(*z*, *k*) is the phase of the measured spectrum while *k* is the distribution of the wavenumbers along the pixels of the camera when using a spectrometer or along the time coordinate when using a swept source. To theoretically describe how axial reflectivity profiles are obtained, we are using continuous variables. However, the reader should be aware that practical implementations involve data digitization, hence variables such as the wavenumber *k* has to be seen as a 1D array containing a number of components equal to the number of sampling points each spectrum is digitized into, in which case *k* is the sampling coordinate (pixel position in spectrometer based interferometry or time in swept-source interferometry). Using a continuous representation of the variables, an axial reflectivity profile *A*(*z*) can be produced by computing:

2 $$A{(z)}^{chirped}={\int }_{-\infty }^{+\infty }E[\phi (z,k)]\cdot {e}^{-jkz}dk$$

where the phase *φ*(*z*, *k*) is expressed as:

3 $$\phi (z,k)=g(k)z+h(k)+{\phi }_{rand}$$

The functions *g*(*k*) and *h*(*k*), present in Eq. 3, contain information on the nonlinear dependence of the phase on the wavenumber and on the dispersion left unbalanced in the interferometer, respectively^9^. *φ*_*rand*_ takes into account random phase shifts introduced between the moment that the system is calibrated and the acquisition moment when the sample is under investigation and the A-scan is produced. In practice, exact values of the wavenumbers *k* do not need to be known, so in the equation above, *g* and *h* can be seen as depending on the position of the pixels on the camera or along time coordinate depending on the SDI method used for spectrum integration. To eliminate the chirping due to nonlinear wavelength mapping and due to unbalanced dispersion, each of the two methods presented here act on different variables. The conventional method widely applied, PCDC operates on experimental spectra while CMS operates on the kernel function. The way in which the two methods are mathematically implemented is described in the following sections.

#### Phase calibration with dispersion compensation (PCDC)

When using PCDC, each experimental acquired spectrum is modified: first resampled then multiplied by a function that cancels the effect of the dispersion. Thus, a linear relationship between the phase of the modified non-chirped spectrum $E(\hat{k})$, and a new wavenumber distribution $\hat{k}$ is obtained. An A-scan compensated for broadening is produced by calculating the FFT of the product between $E(\hat{k})$ and an apodization function $W(\hat{k})$:

4 $$A{(z)}^{non-chirped}={\int }_{-\infty }^{+\infty }W(\hat{k})\cdot E(\hat{k})\cdot {e}^{-jh(\hat{k})}\cdot {e}^{-jkz}dk=FFT[W(\hat{k})\cdot E(\hat{k})\cdot {e}^{-jh(\hat{k})}]$$

The unknown in Eq. 4 is $\hat{k}$. To find it, in PCDC, the effect of the random phase is normally neglected (*φ*_*rand*_ = 0), and a new wavenumber distribution $\hat{k}$ is obtained by polynomial interpolation of the function *g*(*k*) defined as:

5 $$g(k)=\frac{\phi (z,k)-h(k)}{z}$$

To obtain *g*(*k*), two spectra are experimentally recorded for two OPD values between the arms of the interferometer, *E*(*z*_1_, *k*) and *E*(*z*_2_, *k*), and their corresponding phases *φ*(*z*_1_, *k*) and *φ*(*z*_2_, *k*) computed. Thus, information on the nonlinearity of the phase:

6 $$g(k)=\frac{\phi ({z}_{2},k)-\phi ({z}_{1},k)}{{z}_{2}-{z}_{1}}$$

and on the unbalanced dispersion:

7 $$h(k)=\phi ({z}_{1},k)-g(k){z}_{1}$$

is inferred. By using Eq. 5, a new distribution of the wavenumbers $\hat{k}$ and a new function $h(\hat{k})$ are determined. The process of calibration (computing $\hat{k}$ and $h(\hat{k})$) is performed before measurements are carried out. To produce accurate information on the reflectivity from within the sample, each acquired experimental spectrum is resampled according to $\hat{k}$, typically via a cubic B-spline interpolation^25^, to produce $h(\hat{k})$, then multiplied by ${e}^{-jh(\hat{k})}$ to correct for the unbalanced dispersion (Eq. 4). The resampling operation is applied to each acquired experimental spectrum.

Alternatively, the PCDC method can be implemented in a slightly different way. Instead of multiplying resampled spectra $E(\hat{k})$ by the phase factor versus the new coordinate $\hat{k}$, ${e}^{-jh(\hat{k})}$, we can first compute the product *E*(*k*) ⋅ *e*^−*jh*(*k*)^, then resample the result. The first solution is more time efficient than the second one. In the first scenario, after data acquisition, only resampling of *E*(*k*) is required, while ${e}^{-jh(\hat{k})}$ is that computed before data acquisition, at the calibration step. The second scenario requires manipulation of a complex form, hence resampling of the real and imaginary parts of *E*(*k*) ⋅ *e*^−*jh*(*k*)^. The FFT results should be the same, if there are no errors due to resampling. No significant differences were noticed between each of the values of the axial resolution or sensitivity when using these two approaches, while the processing time is longer by a factor around 2 when the resampling of the whole product *E*(*k*) ⋅ *e*^−*jh*(*k*)^ is performed. Therefore we opted for the procedure where resampling is needed for *E*(*k*) only.

#### Complex Master Slave (CMS)

In contrast to the PCDC method, the process of obtaining an axial reflectivity profile utilizing the CMS method consists in modifying the kernel function *e*^−*jkz*^ in Eq. 2. As demonstrated in Rivet *et al*.^18^, an accurate reflectivity profile can be produced by computing:

8 $$A{(z)}^{non-chirped}={\int }_{-\infty }^{+\infty }E[\phi (z,k)]\cdot W(k)\cdot \frac{\partial g}{\partial k}\cdot {e}^{j\phi (z,k)}dk={\int }_{-\infty }^{+\infty }E[\phi (z,k)]\cdot {T}^{\ast }[\phi (z,k)]dk$$

At the calibration step (Master), functions T^*^, referred to from now on as (complex) masks or theoretically inferred spectra,

9 $${T}^{\ast }[\phi (z,k)]=W(k)\cdot \frac{\partial g}{\partial k}{e}^{j\phi (z,k)}$$

are evaluated for the desired sequence of axial points along *z*. In contrast to the PCDC method, the random phase shift *φ*_*rand*_ is now taken into account. However, if the derivative of the phase with respect to *k* is calculated, it is reasonable to suppose that ∂*φ*_*rand*_/∂*k* = 0 so that Eq. 3 can be re-written as:

10 $$\frac{\partial \phi (z,k)}{\partial k}=\frac{\partial g(k)}{\partial k}\cdot z+\frac{\partial h(k)}{\partial k}$$

To compute *T* ^*^, calculations of the phase *φ*(*z*, *k*) as well as of the derivative of *g* with respect to *k* are required. To this goal, as for the PCDC method, two experimental spectra recorded for two different OPD values, *E*(*z*_1_, *k*) and *E*(*z*_2_, *k*) are collected. Then, using Eq. 10, the derivative of *g* and *h* with respect to *k* can be calculated:

11 $$\frac{\partial g(k)}{\partial k}=\frac{1}{{z}_{2}-{z}_{1}}\cdot [\frac{\partial \phi ({z}_{2},k)}{\partial k}-\,\frac{\partial \phi ({z}_{1},k)}{\partial k}]$$

12 $$\frac{\partial h(k)}{\partial k}=\frac{\partial \phi ({z}_{1},k)}{\partial k}-\,\frac{\partial g(k)}{\partial k}\cdot {z}_{1}$$

The functions *g*(*k*) and *h*(*k*) are then computed by integrating Eqs 11 and 12. In this way, a set of complex masks *T* ^*^(*z*_*q*_) is produced, where *q* = 1…*Q*, with *Q* the number of axial points targeted in the A-scan. To produce an A-scan from a sample, after acquiring a spectrum, according to Eq. 8, *Q* dot products between *E*[*φ*(*z*, *k*)] (containing the sample information) and the complex masks *T* ^*^(*z*_*q*_) are calculated. The calibration step involves some mathematical calculations that are not time expensive, and need to be executed once for a given experimental set-up. Let us consider that the goal is to produce an A-scan of *Q* axial points. The PCDC method involves at least three sequential operations to produce each A-scan:

1. Cubic B-spline interpolation of each spectrum.
2. Multiplication of the result by ${e}^{-jh(\hat{k})}$.
3. FFT of the result obtained at step 2.

Apart from these three operations, apodization of data, before interpolation, is commonly needed as well. All these operations are performed sequentially. In contrast to PCDC, the *Q* dot product operations in the CMS can be executed in parallel.

**Figure 12:**
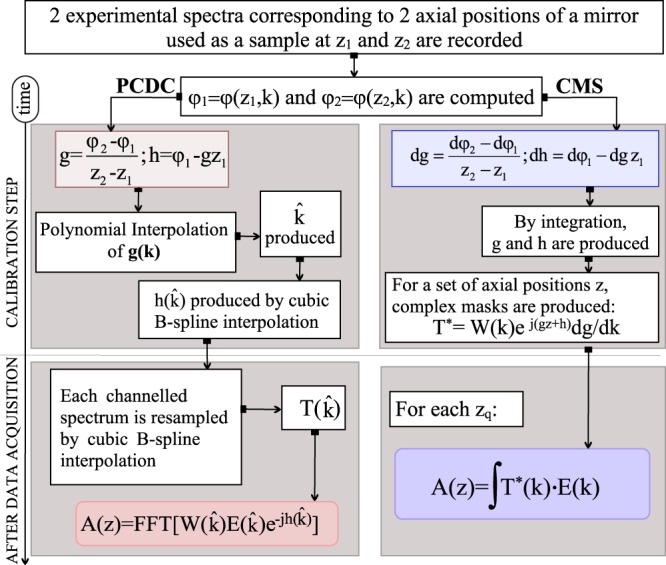
Flowchart showing the steps required by each method at the calibration stage and the mathematical operations to be performed on each spectrum once acquired using the PCDC (left) and CMS (right) methods.

A summary of the mathematical operations required to produce an A-scan using both approaches is shown in Fig. 12. After data acquisition, to produce an A-scan, the PCDC method requires resampling of each experimentally acquired spectrum, its multiplication by ${e}^{-jh(\hat{k})}$ and FFT of the result. The CMS requires, for each depth, a dot product between the corresponding complex mask and the experimental spectrum. According to the above, the PCDC leads to a full A-scan while the CMS leads to a single point of the A-scan. This may look as disadvantageous for the CMS, but it is exactly this property that allows direct production of *en-face* images in OCT, as the PCDC requires the extra step of decomposing all the A-scans to reach the depth of each *en-face* map. Also, as reported in^19^, matrix multiplications allow production of CMS operations in parallel. In sensing, using CMS it may be possible when tracking a moving mirror to perform dot products with a reduced set of masks around the previous position of the mirror. This simplifies the calculations and reduces the time needed. Using the PCDC, A-scans along the whole axial range are produced each time, where clearly the only significant values are those around a single peak. All values outside the region of the peak are not needed.

The sequential mathematical operations required by PCDC (resampling operations followed by FFT) cannot be summarised within a single matrix which eventually is multiplied with a spectrum vector. In contrast, in CMS, the complex matrix T contains theoretically inferred spectra (produced at the calibration step). Using this matrix, as explained in^18^, to obtain an axial reflectivity profile, this complex matrix is multiplied with the acquired spectrum, E:

13 $$A{(z)}^{CMS}=|T\cdot E|=|(\begin{array}{cccc}T({z}_{1},{k}_{1}) & T({z}_{1},{k}_{2}) & \cdots  & T({z}_{1},{k}_{N})\\ T({z}_{2},{k}_{1}) & T({z}_{2},{k}_{2}) & \cdots  & T({z}_{2},{k}_{N})\\ \vdots  & \vdots  & \ddots  & \vdots \\ T({z}_{Q},{k}_{1}) & T({z}_{Q},{k}_{2}) & \cdots  & T({z}_{Q},{k}_{N})\end{array})\cdot (\begin{array}{c}E({k}_{1})\\ E({k}_{2})\\ \vdots \\ E({k}_{N})\end{array})|$$

where each acquired spectrum is sampled into *N* points and each A-scan contains *Q* axial points. The matrix T, is produced using two experimental calibration spectra, and incorporates all the effects determining their chirping (non-linearities and unbalanced dispersion). Each row of the matrix T is a theoretically inferred spectrum corresponding to a specific optical path difference between the arms of the interferometer. The number of the rows in T determines the number of axial points to be computed along a specific axial range not determined by the sampling depth of the digitizer as it is the case with the FFT based methods. If a single row from the matrix T is multiplied with a matrix containing experimental spectral collected at *P* lateral positions while the light is scanned over the sample, then a transversal reflectivity profile (R-scan) from a constant depth *z*_*i*_ is obtained:

14 $$R{({z}_{i})}^{CMS}=|E\cdot T({z}_{i})^{\prime} |=|(\begin{array}{cccc}E({x}_{1},{k}_{1}) & E({x}_{1},{k}_{2}) & \cdots  & E({x}_{1},{k}_{N})\\ E({x}_{2},{k}_{1}) & E({x}_{2},{k}_{2}) & \cdots  & E({x}_{2},{k}_{N})\\ \vdots  & \vdots  & \ddots  & \vdots \\ E({x}_{P},{k}_{1}) & E({x}_{P},{k}_{2}) & \cdots  & E({x}_{P},{k}_{N})\end{array})\cdot (\begin{array}{c}T({z}_{i},{k}_{1})\\ T({z}_{i},{k}_{2})\\ \vdots \\ T({z}_{i},{k}_{N})\end{array})|$$

where *T*(*z*_*i*_)′ represents the transpose of T. To a certain extent, in terms of mathematical operations performed after data acquisition, the CMS method has similarities with the NUDFT based methods, where for a Vandermonde matrix, D needs to be computed at the calibration step. However, there is a fundamental difference between D and T, hence between the NUDFT and CMS. The Vandermonde matrix, also constructed at the calibration step, requires an accurate calibration pixel/wavenumber (i.e. the distribution of the wavelengths along the pixels of the line camera or along time has to be known). D does not include any information on the unbalanced dispersion in the interferometer, hence the NUDFT technique is only applicable to interferometers with perfect dispersion compensation. To obtain an axial reflectivity profile in NUDFT, according to^15^, matrix D is multiplied with the acquired experimental spectrum vector, E:

15 $$A{(z)}^{NUDFT}=|D\cdot E|=|(\begin{array}{cccc}1 & 1 & \cdots  & 1\\ {P}_{1}^{1} & {P}_{2}^{1} & \cdots  & {P}_{N}^{1}\\ {P}_{1}^{2} & {P}_{2}^{2} & \cdots  & {P}_{N}^{2}\\ \vdots  & \vdots  & \ddots  & \vdots \\ {P}_{1}^{N} & {P}_{2}^{N} & \cdots  & {P}_{N}^{N}\end{array})\cdot (\begin{array}{c}E({k}_{1})\\ E({k}_{2})\\ \vdots \\ E({k}_{N})\end{array})|$$

where, the complex parameters *P*_*i*_ are described by:

16 $${P}_{i}=\exp (2\pi j\cdot \frac{{k}_{i}-{k}_{min}}{{k}_{max}-{k}_{min}})$$

In the equation above, *i* = 1…*N*, while *k*_*min*_ and *k*_*max*_ are the limits of the spectral range employed. NUDTF needs information on the distribution of wavelengths along pixels which has to accurately be provided, while CMS infers this distribution as well as the dispersion in the interferometer from a single calibration procedure. A similar procedure to generate the distribution of wavelengths along pixels can complement the NUDFT based method, in which case, each *k*_*i*_ can be replaced by *g*(*k* = *i*), where *k*_*min*_ = *g*(1) and *k*_*max*_ = *g*(*N*).

### Experimental set-up

**Figure 13:**
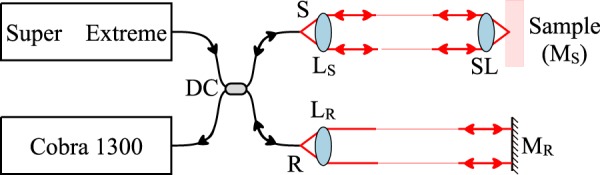
Schematic diagram of the system employed for this study. *L*_*R*_, *L*_*S*_: achromatic lenses; SL: telecentric lens; *M*_*R*_: flat reference mirror. For calibration purposes, the sample is replaced with a flat sample mirror identical to *M*_*R*_ (*M*_*S*_).

A schematic diagram of the UHR spectrometer based SDI instrument used for our experiments is depicted in Fig. 13. Light from a supercontinuum broadband light source (SuperK Extreme, NKT Photonics, repetition rate 320 MHz)^32^ is directed towards the sample (S) and the reference (R) arms of an interferometer by a 50/50 directional coupler DC. In the sample arm, S, light is conveyed towards the sample via an achromatic lens *L*_*S*_, and an achromatic telecentric scan lens SL. When the instrument was used for imaging purposes, a galvanometer mirror was placed between *L*_*S*_ and the sample. At the calibration step, as sample, a flat mirror *M*_*S*_ was employed.

Light back-scattered by the sample and reflected by the reference mirror *M*_*R*_ interferes at DC where it is conveyed towards a commercial spectrometer (Cobra 1300, Wasatch Photonics) that covers a large spectral range, from 1070 to 1470 nm. The spectrometer is equipped with a Sensors Unlimited GL2048 linear InGaAs camera, of 2048 pixels and runs at 76 kHz. The difference between the two arms is required by either the OCT practice, or by sensing, where the telecentric lens and sample mirror in OCT are replaced by a corner cube for measurement of distances (sensing applications). Using the elements normally employed by OCT does not reduce the generality of the study. The spectrometer allows for an axial range of maximum 2 mm to be covered.

The imaging instrument has been previously used in clinical studies (Mogensen *et al*.^33^). The in-house developed OCT imaging instrument described above was used to collect data from optical phantoms as well as for *in-vivo* imaging of the healthy human thumb of one volunteer (co-author, Niels Møller Israelsen). Informed consent was obtained from the volunteer. This instrument was used for a larger clinical study including healthy volunteers from the Department of Dermatology at Bispebjerg Hospital, University of Copenhagen, study established in accordance with Helsinki II Declarations. All methods employed for imaging were performed in accordance with guidelines and regulations as described in the research protocol approved by the Ethics Committee of The Capital Region of Denmark, no. H-16039077.

## Electronic supplementary material

Supplementary material

## Data Availability

The datasets generated and/or analysed in the current paper are available from the corresponding author on reasonable request.
