# Supplementary material for: Recovering distance information in spectral domain interferometry
Source: Sci Rep. 2018 Oct 18;8:15445. doi: 10.1038/s41598-018-33821-0 (PMC6194011; doi:10.1038/s41598-018-33821-0)
Supplement: Supplementary file 1 — Supplementary material [file 41598_2018_33821_MOESM1_ESM.pdf]

# Recovering distance information in spectral domain interferometry

**Adrian Bradu[1,*], Niels Møller Israelsen[2], Michael Maria[2], Manuel J. Marques[1], Sylvain Rivet[3], Thomas Feuchter[4], Ole Bang[2,4], and Adrian Podoleanu[1]**

[1]Applied Optics Group, School of Physical Sciences, University of Kent, CT2 7NH Canterbury, UK
[2]DTU Fotonik, Department of Photonics Engineering, Technical University of Denmark, DK-2800 Kongens Lyngby, Denmark
[3]Laboratoire d'Optique et de Magnétisme EA938, IBSAM, Univ. Bretagne Occidentale, C.S. 93837, 29238 Brest Cedex 3, France
[4]NKT Photonics A/S, Blokken 84, DK-3460 Birkerod, Denmark
[*]Corresponding author: a.bradu@kent.ac.uk

## ABSTRACT

This work evaluates the performance of the Complex Master Slave (CMS) method, that processes the spectra at the interferometer output of a spectral domain interferometry device without involving Fourier transforms (FT) after data acquisition. Reliability and performance of CMS are compared side by side with the conventional method based on FT, phase calibration with dispersion compensation (PCDC). We demonstrate that both methods provide similar results in terms of resolution and sensitivity drop-off. The mathematical operations required to produce CMS results are highly parallelizable, allowing real-time, simultaneous delivery of data from several points of different optical path differences in the interferometer, not possible via PCDC.

## Supplementary material

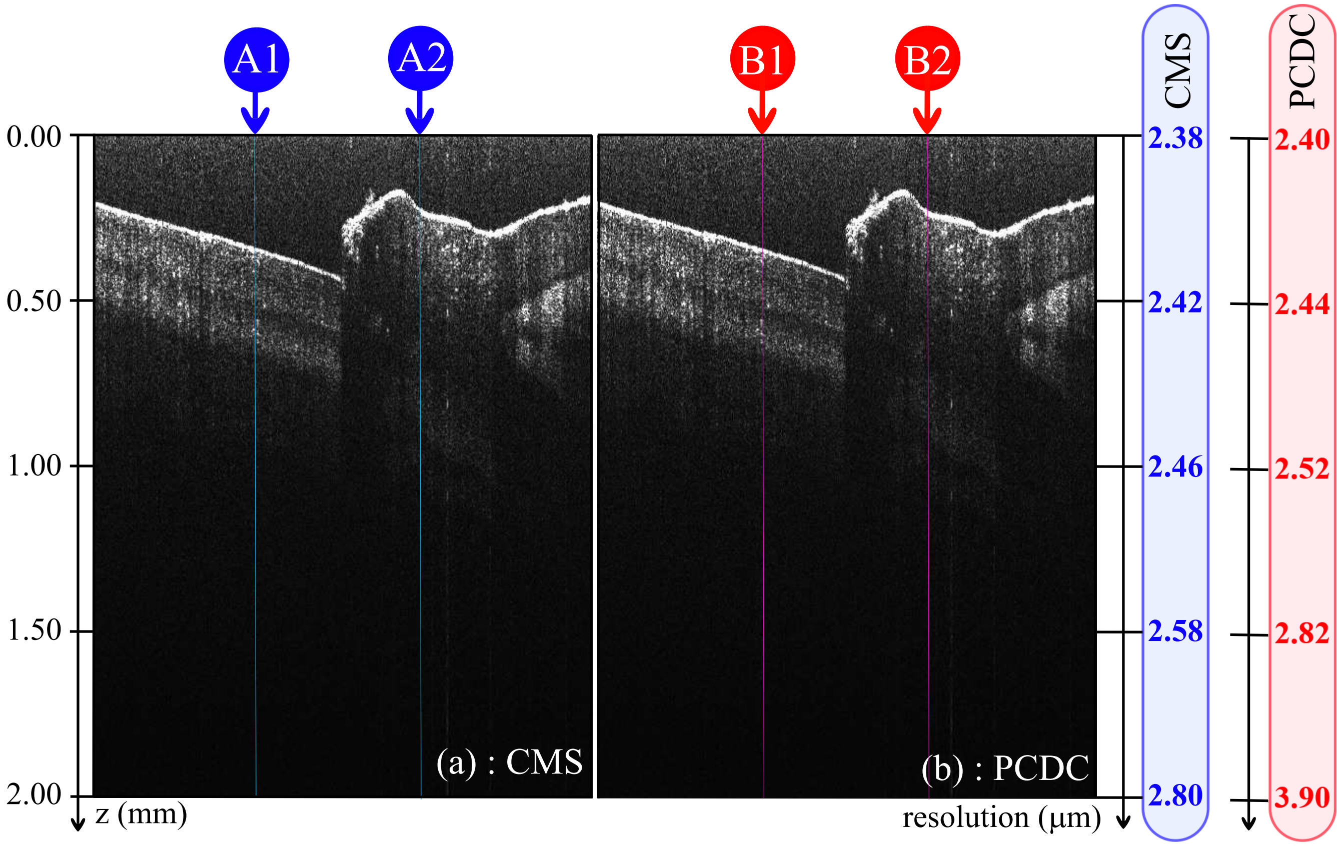


**Supplementary Figure S 1.** Cross-section images from the junction nail/skin a thumb of a volunteer obtained using CMS (a) and PCDC (b). The left vertical axis is depth *z*, the right vertical axes are the axial resolutions as measured in air using both methods.

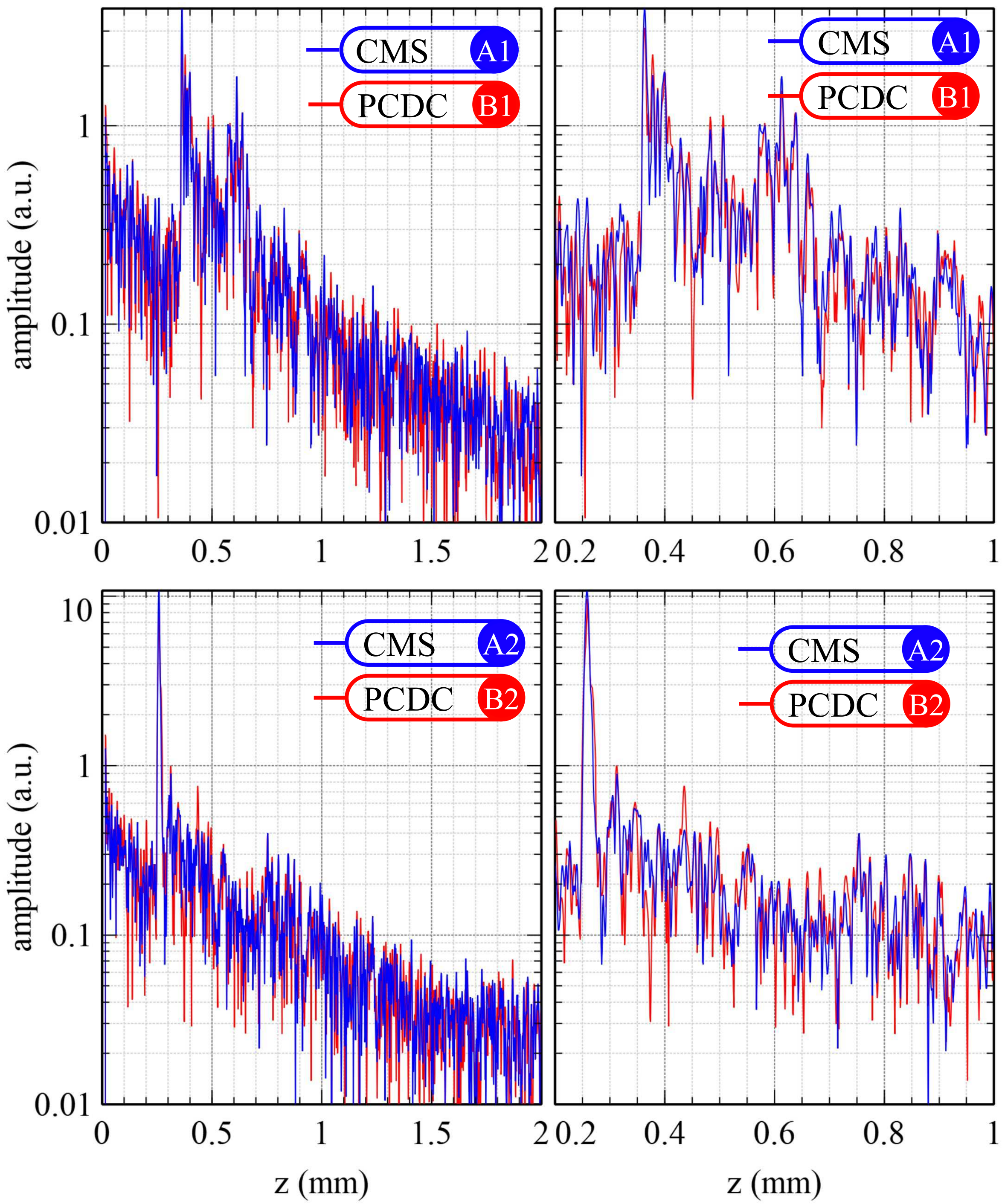


**Supplementary Figure S 2.** A-scans produced using PCDC and CMS from the junction skin/nail for the entire axial range from 0 to 2 mm in the left figure and for the 0.2-1 mm axial range in the right. Data were extracted from the images shown in Fig. S1 at the positions marked by a blue dashed line A1 and A2 (CMS) and red dashed line B1 and B2 (PCDC).
